# A Medical Bibliographic Source of the Iran–Iraq War (1980–1988)

**DOI:** 10.34172/aim.2023.71

**Published:** 2023-08-01

**Authors:** Mohammad Hossein Azizi

**Affiliations:** ^1^Academy of Medical Sciences of the I.R. of Iran, Tehran, Iran

**Figure 1:**
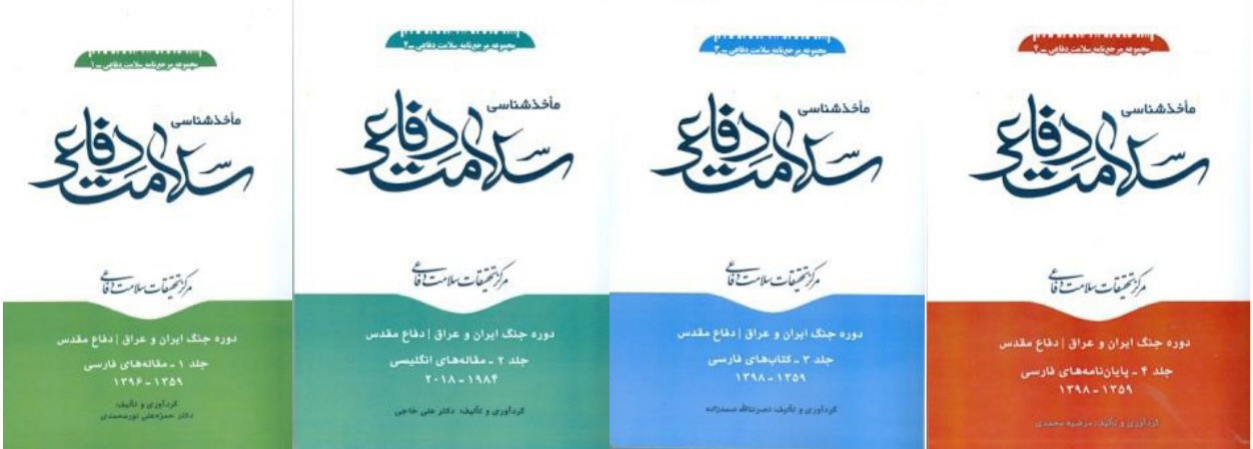
Back Covers of the Four-volume Set of Medical Bibliographic Books of the Iran–Iraq War (1980–1988), Published in 2023

 Recently in May 2023, a four-volume set of medical bibliographic collection of the Iran –Iraq war (1980-1988), including English- and Persian-language scientific papers, books and dissertations was published (Figure 1).^1^

 This collection consists of the following bibliographic books:

**Volume 1** contains 894 Persian-language articles initially published between 1980 -2017.

**Volume 2** covers 602 English-language papers printed between 1980-2018.

**Volume 3** includes 509 Persian-language books published between 1980 -2019.

**Volume 4** consists of 1393 Persian-language dissertations pertaining to 1980-2019.

 According to the research supervisor of this collection, Jalil Arab Kherdmand MD, the existing English-language data in the second volume of the above-mentioned four-book series was collected from various databases including the PubMed, Scopus, Web of Science, Cochrane, EMBASE and Google Scholar by the research team.

 Essentially, human wars are destructive and damaging and cause mortality and morbidity, and occasionally its physical and psychological consequences remain for years in victims. Thus, medical researchers have been sharing their medical observations and management approaches from previous military conflicts. For instance, by searching the words “First World war”, “Second World War”, “US Vietnam War”, “War in Afghanistan” and “Iran-Iraq war”, 5024, 9451, 369, 2879 and 333 papers are found respectively on the PubMed database until 3.6.2023.

 In these medical works, the investigators discussed various aspects of war causalities and have described their experiences scientifically, in particular to other health professionals and surgeons in various fields. Chemical weapons were another disastrous issue of some wars in the past decades which are challenging for toxicologists, dermatologists, neurologists, ophthalmologists, internists and pulmonologists. In addition, the psychological impact of the military conflicts on both armed forces and civilians is a substantial matter. Publishing the scientific medical experiences of wars is also beneficial for medical management of other injured people during natural disasters such as earthquakes, floods and damages due to motor vehicle accidents and occupational injuries.

 In the recent medical bibliographic book in English (Vol. 2), Compiled by Dr. Ali Khaji which is devoted to the Iran-Iraq war (1980-1988), some important medical and surgical topics have been written in English and published in local or international health periodicals by both Iranian specialists and some experts from other countries. Overall, these 602 articles address different aspects of medical, surgical, psychological, and nursing care. Among chemical-warfare agents, mustard gas was widely used by the Iraqi forces against Iranian troops and civilians especially in Western Iran, and information on early and late complications of the eyes, skin and lungs are provided.

 In conclusion, the current bibliographic collection is an important and informative medical source of health professional experiences of the Iran-Iraq war which helps other researchers with further investigations. The subjects, article titles and author’s name indices of the book are likewise very useful.

 Finally, I suggest the publication of a single-volume book selecting the best works in this four-book series, by taking into account the quality of articles based on the papers’ citations, impact factor of the journals in which they are published, as well as the novelty and methodology of the written papers. Improving the layout and editing the English version are desirable goals in order to eliminate some typo errors, providing a more uniform articles’ titles format and correcting potential deficits.
